# Synopsis Annual Meeting Illinois State Dental Society

**Published:** 1884-07

**Authors:** 


### PROCEEDINGS.

### Synopsis of the Annual Meeting of the Illinois State Dental Society.

The Society met May 13th, at 10 o'clock a.m., in the State house, at Springfield. The President, Dr. E. Noyes, of Chicago, in the chair.

The meeting opened by prayer by the Rev. R. O. Post. After some miscellaneous business, the reports of the Executive Committee, Treasurer, and Secretary, were presented.

The President now delivered his annual address, which contained much valuable information. His suggestions in regard to the special work of the Association were highly appreciated; and in order to bring them fully into operation a committee of three, consisting of Drs. Cushing, Kitchen, and Black, were appointed to report to the Society. Papers were called for, but none were at hand, they were promised for the afternoon. The initiation fee was reduced from $7 to $5. The Standard of admission was raised. The Examining Committees were instructed to make their work conform to this requirement.

An invitation was received to visit Lincoln's monument, at the the convenience of the Society. The invitation was accepted.

Adjourned.

### Afternoon Session.

The meeting opened at the usual hour; and after miscellaneous business, a paper was read by Dr. Duncan, on "Exposed Pulps." The paper was discussed by Drs. Crouse, of Chicago, Patrick, Townsend, Nelson, and Eames. These discussions occupied the whole afternoon.

Adjourned.

### SECOND DAY—Morning Session.

The Society met at 9:30 with a greatly increased attendance, there being over 100 active members present, and in addition a large number of visitors.

The minutes of the previous meeting were read, after which the following persons were elected to serve upon the Executive Committee for the following year; namely, Dr. Pollock, of Sterling, Chairman, and Dr. C. F. Mattison, of Chicago. The third member to be chosen later.

Mrs. Dr. C. K. Moody, of Mendota, read a paper on "Reflex Pain." The paper was one evincing much thought and study. It showed that the author was quite familiar with her subject. She spoke of various "nerve affections." As to remedies she spoke highly of gelseminum, in five to twenty drop doses, for direct pain, from the branches of the fifth pair, and quinine for "reflex pain." She referred at some length to the various causes of "reflex pain."

In discussion of the paper, Dr. Freeman, of Chicago, cited a few cases. The first was a case of "reflex pain," from a tooth, upon the uterus.

The lady came to him, having all her teeth quite loose, and desired them extracted. He thought upon the case for a few days; consulted her husband, and refused to remove the teeth. In three months the teeth were firm, and there was a complete return of health.

In another case a little child, when teething, had persistent diarrhœa for a long time. He prescribed laudanum and castor oil. The bowels were restored to healthy action. Cohrea set in, they hoped that this would soon pass away, having incised the gums.

In another case the patient had some amalgam fillings. Some considerable time afterward the masseter muscle became constricted and rigid. A magnetic doctor examined the case, and removed the third molar tooth; which seemed to be the cause when the difficulty was entirely relieved.

Dr. G. V. Black regards neuralgia as occurring from perverted "vaso motor" action, in which there is interrupted blood supply to the part. This causes a nerve tension, which results in neuralgia.

Dr. Patrick regards Jamaica dogwood, ten to twenty drops, as preferable to gelseminum; regards it as safer and better.

The paper was further discussed by Drs. Townsend, Sitherwood, and Noyes.

Prof. Eames, of St. Louis, read a paper on the "Origin of Defective Tooth Structure." The paper was one of great interest, and pursued a line of thought eminently practicable. The subject is one that ought to have received more attention on the part of the profession than it has hitherto.

Miscellaneous business was now called. Dr. Black moved that a committee of three be appointed to take into consideration the propriety of establishing an association journal, to be controlled and managed jointly by the "Illinois and Chicago Dental Societies."

Adjourned.

### Afternoon Session.

The Society met at 2:30. After some routine business, four new members were elected, as follows: A. E. Burrows, of Walnut; F. H. McIntosh, of Bloomington: Robt. Gaebel, of Lincoln; R. H. Mace, of Belleville.

Dr. Dean, of Des Moines, Iowa, was elected an honorary member.

A motion was offered and passed to reconsider a resolution recommending a beneficiary student to Baltimore Dental College. Subject was then referred to a committee of Examiners.

Dr. Homer Judd, of Alton, read a paper, which was very interesting. Subject, "Inflammation," which called forth considerable discussion.

An invitation was extended to the members of the Association, by Mr. Oldroyd, to visit Lincoln's old home, which was accepted, and the visit was made at 5 o'clock, after adjournment. This proved to be a very interesting occasion, as a great many relics of his home and time are there kept; many things to call to memory the time from 1860 to 1864.

### Evening Session.

The Association met at 8 o'clock. After some routine business, Dr. Will. X. Sudduth, of Bloomington, read a paper on "Dento-Embryonal Histology," which attracted special attention. The paper was discussed somewhat at length, but not in a manner to be reported.

Miscellaneous business was now taken up, when arrangements were made for clinics from 8:30 to 12:30 to-morrow.

Adjourned.

### THIRD DAY—Morning Session.

The entire morning was devoted to clinics, in which there were some ten or twelve operators. Various styles of filling were demonstrated, and different methods of inserting artificial crowns.

Dr. J. A. Swasey, of Chicago, illustrated his method of filling in an operation on a left inferior molar with a large and complicated cavity. He used Robinson's fibrous filling, finishing with gold foil, Nos. 4 and 10. The operation was made for the purpose of showing that the combination of different materials makes a better filling in teeth with frail walls, and that require large contour, than either material alone.

Dr. James G. Reid, of Chicago, demonstrated his method of filling roots with a preparation of gutta percha dissolved in chloroform. He selected the first inferior left molar. Owing to its unfavorable situation it was a difficult tooth to operate on. The moisture was not easily excluded. The result was an entirely satisfactory operation. This is not a new method of filling roots, but Dr. Reid showed great skill and facility in the performance of the operation. This is regarded as one of the best methods of filling roots that has ever been devised.

Dr. E. Honsinger, of Chicago, operated on an inferior second molar posterior cavity. Watts' crystal gold was used, and over two hours time was occupied. The result was a beautiful operation, and one that seemed in all respects well nigh perfect.

Dr. H. H. Townsend, of Pontiac, operated upon a superior left first bicuspid, from which the pulp had been removed. The canal was filled with gutta percha. The cavity was upon the posterior, and across the grinding surface. The operation was made with great care, and presented an excellent appearance.

Dr. E. D. Swain, of Chicago, operated on an inferior molar. The crown of which extended considerably below the margin of the gum. The tooth was restored with care to its original shape and size.

It was very gratifying to know that in each of these operations, and, indeed, in nearly all the operations performed, there was a precision and delicacy of manipulation that was very gratifying indeed. There is evidently in the profession a growing recognition of the fact that more care and skill in manipulation is required than was formerly thought necessary. The clinic closed at 1 o'clock.

AFTERNOON SESSION.

The Association met at 2:30. Miscellaneous business was in order. On motion, Dr. Judd was reinstated to active membership with remission of annual dues.

The first business in regular order was the reading of a paper by Dr. Taggart on "Chemistry." The Doctor had on his table quite an array of chemical apparatus. By actual experiment he was able to illustrate the points presented in his paper. He made tests of a number of medicinal agents in daily use by the dentists, such as arsenious acid, creosote, carbolic acid, chloroform, ether, etc. He described a method of preparing oxyphosphate of zinc, for plastic filling. The paper with the illustrations given contains a large amount of valuable and interesting matter.

Some discussion was had upon the paper. It took the form, however, more of a quiz of Dr. Taggart, with the view of bringing out some points that had not been understood by some of the members. This was rather an innovation upon the ordinary method of discussion, but of course it was perfectly admissible, and perhaps if there was more questioning under such circumstances, there would be a better general appreciation of what was presented than is many a time shown.

Dr. Black presented a paper on "Specialists and Specialties." This, as is every thing from Dr. Black's pen, was very interesting, and received the closest attention on the part of the members. After this, Dr. Patrick delivered a brief address on "A Few Stray Leaves from Dental Education." The committees, Drs. Stone and Koch, on New Dental Appliances and Inventions, reported that quite a large number had been presented. They then proceeded to bring them before the Society, explaining and making clear the use of each one. There was quite a number of new and valuable instruments, etc.

Adjourned.

### Evening Session.

The evening was devoted to the representation, with the stereopticon, of specimens illustrating dento-embryology, by Dr. Will. X. Sudduth, of Bloomington. About seventy-five illustrations were thrown upon the screen, which received proper explanation. About three hours were occupied in this work. To Dr. Will. X. Sudduth is due the thanks and appreciation of the profession for the very thorough and efficient work he has accomplished in this direction This method of illustration should be used far more than it is. Many people comprehend more readily by an appeal to the eye than to the ear.

Adjourned.

### FOURTH DAY—Morning Session.

After routine business, Dr. R. E. Koch, of Chicago, read a paper entitled "The Illinois Dental Society—What It has Accomplished." The paper gave the history of the organization, told of its achievements, and showed that it has been a power for good in the State of Illinois, and also that its influence had been felt outside of the State. He thought that all the members were justified in feeling a gratification for all that has been done by the body.

On motion of Dr. Black,

*Resolved*, That a supervisor of clinics be appointed whose duty it shall be to arrange for and have control of the clinics held at the annual meeting. He shall also make a report upon past clinics, with the view of illustrating the progress attained.

The election of officers for the ensuing year was now declared to be in order, which being had, Dr. H. H. Townsend, of Chicago, was elected President; Dr. M. H. Patton, of Springfield, Vice-President; Dr. J. W. Wassall, of Chicago, Secretary; Dr. Dwight, of Danville, Treasurer.

The next annual meeting is to be held at Peoria. A vote of thanks was extended to various parties, namely, proprietors of the Leland Hotel, railroad companies, and Secretary of State, Mr. Dement, for the use of the Senate Chamber for the meetings.

Adjourned.

